# Visual search behavior and decision-making in expert and less-experienced football referees across different foul situations: an eye-tracking study

**DOI:** 10.3389/fpsyg.2026.1871146

**Published:** 2026-06-10

**Authors:** Zilu Zhou, Rancheng Tao, Kongyun Huang, Changjing Zhou

**Affiliations:** 1 School of Football, Shanghai University of Sport, Shanghai, China; 2 Complex Systems Group & GISC (Grupo Interdisciplinar de Sistemas Complejos), Universidad Rey Juan Carlos, Móstoles, Spain

**Keywords:** expertise differences, eye movement, football referee, foul judgement, perceptual–cognitive expertise, visual search

## Abstract

**Introduction:**

Modern football imposes considerable demands on referees' rapid foul judgements, yet the dissociation between accuracy and efficiency of officiating expertise across different foul situations remains poorly characterized.

**Methods:**

The present study compared 15 national-level and 15 Class-3 male football referees in three typical situations: ball-contesting, tactical foul and handball. Participants viewed 30 real-match video clips and made one technical and one disciplinary decision per clip while an EyeLink 1000 Plus eye tracker recorded global eye-movement indices and area-of-interest (AOI) measures. Data were analyzed with 2 (group) × 3 (situation) mixed-design ANOVAs and independent-samples *t*-tests.

**Results:**

Decision accuracy did not differ between groups for either technical or disciplinary judgements, although a robust situation main effect was observed [technical accuracy *F*_(2,56)_ = 116.45, *p* < 0.001, η^2^*p* = 0.81; disciplinary accuracy *F*_(2,56)_ = 13.73, *p* < 0.001, η^2^*p* = 0.33]. For decision efficiency, disciplinary reaction time showed a Group × Situation interaction [*F*_(2,56)_ = 4.16, *p* = 0.021, η^2^*p* = 0.13]; simple-effect analysis indicated that national-level referees responded faster than Class-3 referees in ball-contesting situations [*F*_(1,28)_ = 6.07, *p* = 0.020, η^2^*p* = 0.18]. Pupil diameter showed an analogous interaction (*p* = 0.026), although within-situation simple effects did not reach significance. AOI analyses revealed that national-level referees fixated the upper body of the fouling player earlier in ball-contesting situations [*t*(26.02) = −2.38, *p* = 0.024, *d* = 0.87] and revisited the attacker's lower body and the defender's upper body less often in handball situations (both *p* < 0.05, *d* ≥ 0.80).

**Discussion:**

Within this video-based task, expertise-related differences emerged primarily in efficiency-related indices rather than overall accuracy; sample size, the simulated nature of the stimuli and ceiling-or-near-chance effects in some conditions limit generalization, and the findings warrant validation in real-match settings.

## 1 Introduction

Refereeing decisions safeguard the fairness and continuity of football matches. The increasing pace and physical intensity of modern football have shortened the available decision window, demanding rapid integration of player movement, contact points and the unfolding tactical context (Catteeuw et al., 2009; Helsen et al., 2023). Over the past two decades, research on referees' decision mechanisms has progressively moved from outcome-level accuracy statistics to perceptual–cognitive processing, with eye-tracking technology providing a direct window onto this processing (Kredel et al., 2017, 2023; Ziv et al., 2020).

The relationship between officiating experience and decision performance has been consistently reported across multiple sports. Expert referees typically achieve higher accuracy and shorter reaction times (Mann et al., 2007; Wu et al., 2024), advantages attributed to more efficient information processing and pattern-recognition mechanisms developed through extended practice (Chase and Simon, 1973; Ericsson et al., 1993). At the visual-search level, experts allocate fixations more selectively and steadily, dwell on each fixation for shorter durations, and concentrate attention on task-relevant regions, enabling faster extraction of decision-critical information (Spitz et al., 2016; van Biemen et al., 2022). Although visual-search studies of football referees have addressed foul situations, the majority focus on a single situation type or on global indices, leaving systematic comparisons across foul situations underexplored (Helsen et al., 2023).

A further limitation is that the dissociation between two distinct dimensions of expertise (decision *accuracy* and decision *efficiency*) has not been adequately characterized in the expert–less-experienced framework. Rule knowledge can be acquired through systematic training; intervention studies show that structured judgement training reliably improves less-experienced accuracy on standardized tasks (Put et al., 2013; Zhou et al., 2025), suggesting that accuracy alone may not reliably distinguish expertise levels. In addition, the ceiling effect observed in handball situations and the floor effect observed in ball-contesting situations suggest that stimulus difficulty may have influenced group comparisons in decision accuracy. Therefore, the absence of significant group differences in accuracy should not be interpreted as definitive evidence of equivalent perceptual–cognitive expertise across all situations. By contrast, reaction time, pupil diameter and AOI-based fixation allocation are likely to reflect the perceptual–cognitive advantages accrued through long-term officiating practice (Hancock and Ste-Marie, 2013; Mann et al., 2007). However, how these two dimensions manifest across foul situations (particularly in ball-contesting, tactical foul and handball, which differ markedly in informational cues and judgement criteria) has yet to be examined directly (Samuel et al., 2024; Spitz et al., 2016).

The present study therefore adopted an expert–less-experienced comparative design with eye-tracking to examine national-level and Class-3 football referees in the three target situations. Behavioral performance, global eye-movement indices, AOI-level fixation allocation and scan-path visualization were compared concurrently to identify the dimensions and situations in which expertise-related differences emerge. Drawing on the perceptual–cognitive expertise literature, rule knowledge can be acquired through systematic judgement training (Catteeuw et al., 2009; Put et al., 2013), whereas advantages accrued through extended officiating experience are more likely to surface in efficiency and attention allocation (Hancock and Ste-Marie, 2013; Mann et al., 2007). On this basis, decision accuracy, decision efficiency and AOI-level fixation allocation were treated as multidimensional indicators of refereeing expertise. Based on the perceptual–cognitive expertise literature, it was hypothesized that: (1) decision accuracy would not differ significantly between groups, as rule knowledge can be acquired through systematic training; (2) expert referees would demonstrate greater decision efficiency, reflected in shorter reaction times, particularly in more complex foul situations; and (3) expert referees would exhibit more efficient visual search behavior, including earlier allocation of attention to task-relevant AOIs and fewer revisits, indicating more effective information extraction and processing. In addition, these expertise-related differences were expected to vary across foul situations, reflecting the context-specific nature of perceptual–cognitive processing in refereeing.

## 2 Materials and methods

### 2.1 Participants

Chinese football referees are graded into five tiers (international, national, Class 1, Class 2 and Class 3), differing markedly in the level of competition they may officiate, annual match volume and accumulated training. To maximize the expected effect and improve sensitivity to dimension-specific differences (Mann et al., 2007; Wu et al., 2024), the present study recruited referees from both ends of this hierarchy. Through the official recruitment channel of the Shanghai Referee Committee of the Chinese Football Association, 30 male football referees were enrolled, divided equally into expert and less-experienced groups (15 per group), all aged between 18 and 30 years. The expert group comprised national-level referees registered with the Chinese Football Association (age *M* ± *SD* = 24.8 ± 2.0 years; refereeing experience *M* ± *SD* = 3.7 ± 1.5 years), who officiated in the Chinese Football Association Super League and above with no fewer than 15 matches per year. The less-experienced group comprised Class-3 referees (age *M* ± *SD* = 21.1 ± 0.9 years; refereeing experience *M* ± *SD* = 1.0 ± 0.4 years) who had passed the certification examination and the basic-rules training course, and who principally officiated grass-roots and amateur events in Shanghai (such as district-level leagues and university tournaments). Although these referees had received formal training and certification, they had substantially less officiating experience and match exposure than the national-level referees; therefore, they are referred to as less-experienced rather than novice referees in the present study. All participants were right-handed, had normal or corrected-to-normal vision with no color-vision deficiencies, and were proficient in computer use. Written informed consent was obtained prior to the experiment, and a modest payment was provided upon completion. The study was approved by the Ethics Committee of Shanghai University of Sport (Approval No. 102772025R7165).

### 2.2 Apparatus and stimuli

**Figure 1:**
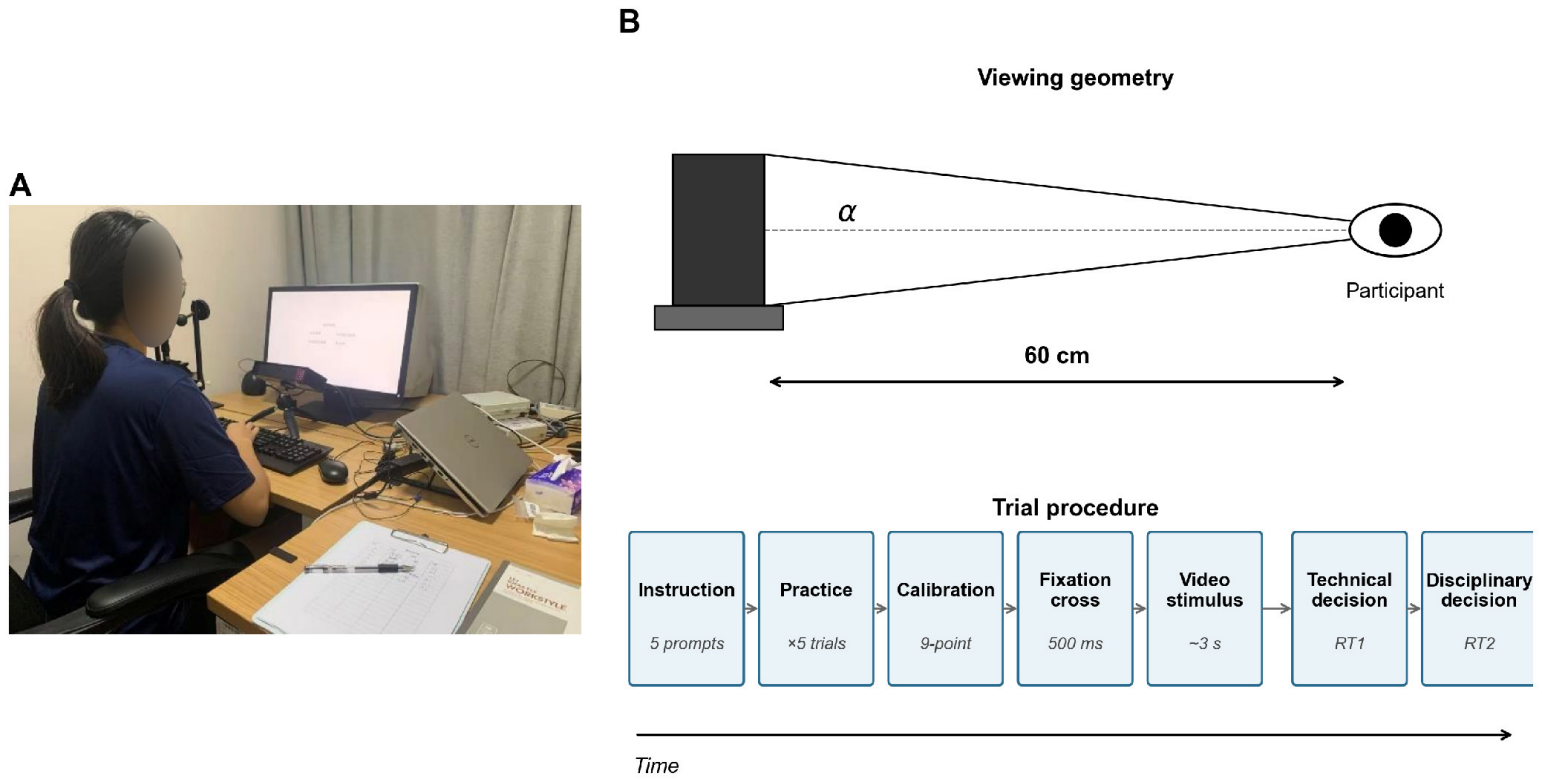
Apparatus and trial procedure. **(A)** Photograph of the experimental set-up; the host computer (left) presented stimuli, while the recording computer (right) acquired eye-movement signals. **(B)** Top: schematic of viewing geometry, with the participant 60 cm from the display; bottom: temporal sequence of a single trial: instruction, five practice trials, calibration, fixation cross (500 ms), video stimulus (~3 s), response.

Eye-movement data were recorded using an EyeLink 1000 Plus eye tracker (SR Research Ltd., Ottawa, Ontario, Canada) at a sampling rate of 1,000 Hz, with an average spatial accuracy of approximately 0.5° (Kredel et al., 2017). Stimuli were presented on a desktop computer, with a separate system synchronously logging eye-movement signals. Participants sat approximately 60 cm from the screen, with the head stabilized by a chin rest to minimize movement artifacts (Figure 1A).

The stimulus set comprised 30 real-match video clips obtained from the FIFA Referee Assistance and Education (FIFA RED) platform, evenly distributed across three foul situations: ball-contesting, tactical foul and handball, with 10 clips per situation (Spitz et al., 2016; van Biemen et al., 2022). Clips were selected based on the following criteria: (1) clear visual quality from a representative broadcast camera angle, (2) clear correspondence to one of the three target foul situations, and (3) availability of an officially adjudicated decision on the platform. In addition, care was taken to ensure that the selected clips were representative of typical match situations encountered in real officiating contexts. No formal pre-evaluation or difficulty-balancing procedure was conducted prior to clip selection. Instead, the clips were selected to represent a range of realistic foul situations commonly encountered in football officiating and to preserve ecological validity. Each clip captured the pre-foul, during-foul and post-foul phases, each lasting approximately 1,000 ms (total duration ≈ 3,000 ms). The criterion decision for each clip was taken from the FIFA RED official adjudication, determined by FIFA-licensed referee instructors in panel review based on the *Laws of the Game*. These adjudications were based on expert consensus and served as the reference standard for evaluating participants' decision accuracy.

Areas of interest (AOIs) were defined for the two situations in which player interaction constitutes the key judgement information. In ball-contesting, AOI1–AOI4 corresponded respectively to the upper body of the fouled player, the lower body of the fouled player, the upper body of the fouling player and the lower body of the fouling player. In handball, AOI1–AOI4 corresponded to the lower body of the attacking player, the upper body of the defending player, the ball and the arm of the defending player. Tactical foul situations were not assigned fixed AOIs because of the highly dynamic positioning and heterogeneous interaction structure; only global eye-movement indices and visualization analyses were retained for this situation. AOIs were defined based on task-relevant regions that are known to provide critical visual information for foul judgement (e.g., contact points, body posture, and ball location). In dynamic scenes, AOIs were adjusted frame-by-frame to follow the movement of players and the ball, ensuring that they consistently captured the relevant regions throughout each clip. The definition of AOIs was informed by previous eye-tracking studies on football referees and perceptual–cognitive expertise (e.g., Spitz et al., 2016; van Biemen et al., 2022), which have identified these regions as critical sources of information for decision-making.

### 2.3 Procedure

The experiment was conducted in a controlled eye-tracking laboratory under low ambient lighting and minimal noise. After being seated, participants received standardized instructions, completed practice trials to familiarize themselves with the procedure, and underwent a nine-point calibration. In the main task, the 30 video clips were presented in randomized order, with the situation type undisclosed in advance. After each clip, participants made a technical decision (no foul, indirect free kick, direct free kick or penalty kick) followed by a disciplinary decision (no card, yellow card or red card) via keyboard input, with no performance feedback provided.

Response keys were mapped consistently across participants. For technical decisions, the keys “A,” “S,” “D,” and “F” corresponded to “no foul,” “indirect free kick,” “direct free kick,” and “penalty kick,” respectively. For disciplinary decisions, the keys “J,” “K,” and “L” corresponded to “no card,” “yellow card,” and “red card,” respectively. Participants first entered the technical decision, followed by the disciplinary decision. Reaction time was automatically recorded by the computer at the moment of keypress and was defined as the interval between the onset of the video stimulus and the participant's response following clip offset. Reaction times were recorded separately for technical and disciplinary decisions.

Eye-movement data were recorded continuously, and the entire session lasted approximately 20 min (Figure 1B).

Behavioral performance was characterized by the decision accuracy of each judgement (ratio of correct responses to total decisions) and the reaction time (defined as the interval between the onset of the video stimulus and the participant's keypress response following clip offset). Global eye-movement indices comprised fixation duration (reflecting cognitive processing load), fixation count (an index of visual processing efficiency), saccade count (reflecting the length and efficiency of search), and pupil diameter (positively associated with cognitive load).

### 2.4 Data analysis

An *a priori* power analysis was conducted at the design stage using G*Power 3.1.9.7 (Faul et al., 2007). On the basis of the effect-size estimates reported by (Wu et al. 2024) for expert vs. non-expert sports officials (decision accuracy *d* = 1.09; fixation count *d* = 0.71), detecting a Group × Situation interaction (the primary inferential target) required a total sample of *N* = 15–28 (α = 0.05, 1 − β = 0.80, Cohen's *f* = 0.25–0.35). The final sample (*N* = 30; 15 per group) thus satisfied the power requirement of the principal analysis and likewise covered the requirement of the AOI independent-samples *t*-tests at typical effect sizes in this literature. Given the limited size of the population of national-level referees, the group main effect (*N* = 86 required at *f* = 0.25) was treated as a secondary analysis; effects below *f* = 0.25 (group main effect) or *d* = 1.06 (independent *t*-tests) are acknowledged to be underpowered in the present design.

Eye-movement data were preprocessed in DataViewer 4.4.1 (SR Research Ltd., Ottawa, Ontario, Canada), including trial segmentation, drift correction, AOI definition and fixation filtering; segments containing blink artifacts or tracking loss, and trials in which drift exceeded 1°, were excluded, and the remaining data were verified by per-participant visual inspection. Per-participant means within each situation were used for subsequent analysis; trial-level values exceeding ±3 *SD* from the participant's mean were removed to mitigate the influence of within-individual extreme responses. The AOI-level indices comprised total fixation duration, fixation count, time to first fixation (interval from stimulus onset to the first fixation within an AOI, reflecting the speed of initial information extraction) and revisit count (returns to a previously fixated AOI, reflecting processing difficulty or re-evaluation). Visualization analyses used fixation heat maps (color intensity corresponding to fixation frequency and duration) and scan-path plots (presenting fixation sequence and saccade direction) to complement the quantitative results.

Statistical analyses were performed in R 4.4.1. Global behavioral and eye-movement indices were submitted to a 2 (group: expert, less-experienced) × 3 (situation: ball-contesting, tactical foul, handball) mixed-design ANOVA. The Greenhouse–Geisser correction was applied to degrees of freedom whenever Mauchly's sphericity test indicated a violation, and the Holm–Bonferroni correction was used for *post-hoc* pairwise comparisons of the situation main effect to control the family-wise error rate. AOI-level group comparisons were conducted with independent-samples *t*-tests; these comparisons were guided by prior research on expert–less-experienced differences in refereeing and targeted theoretically relevant regions; however, given the number of AOIs and comparisons, the results should be interpreted with caution and considered partly exploratory (Spitz et al., 2016; van Biemen et al., 2022) in light of the multiple-comparisons concern. Effect sizes are reported as partial eta-squared ($\eta_{p}^{2}$) or Cohen's *d* throughout.

## 3 Results

### 3.1 Behavioral performance

**Figure 2:**
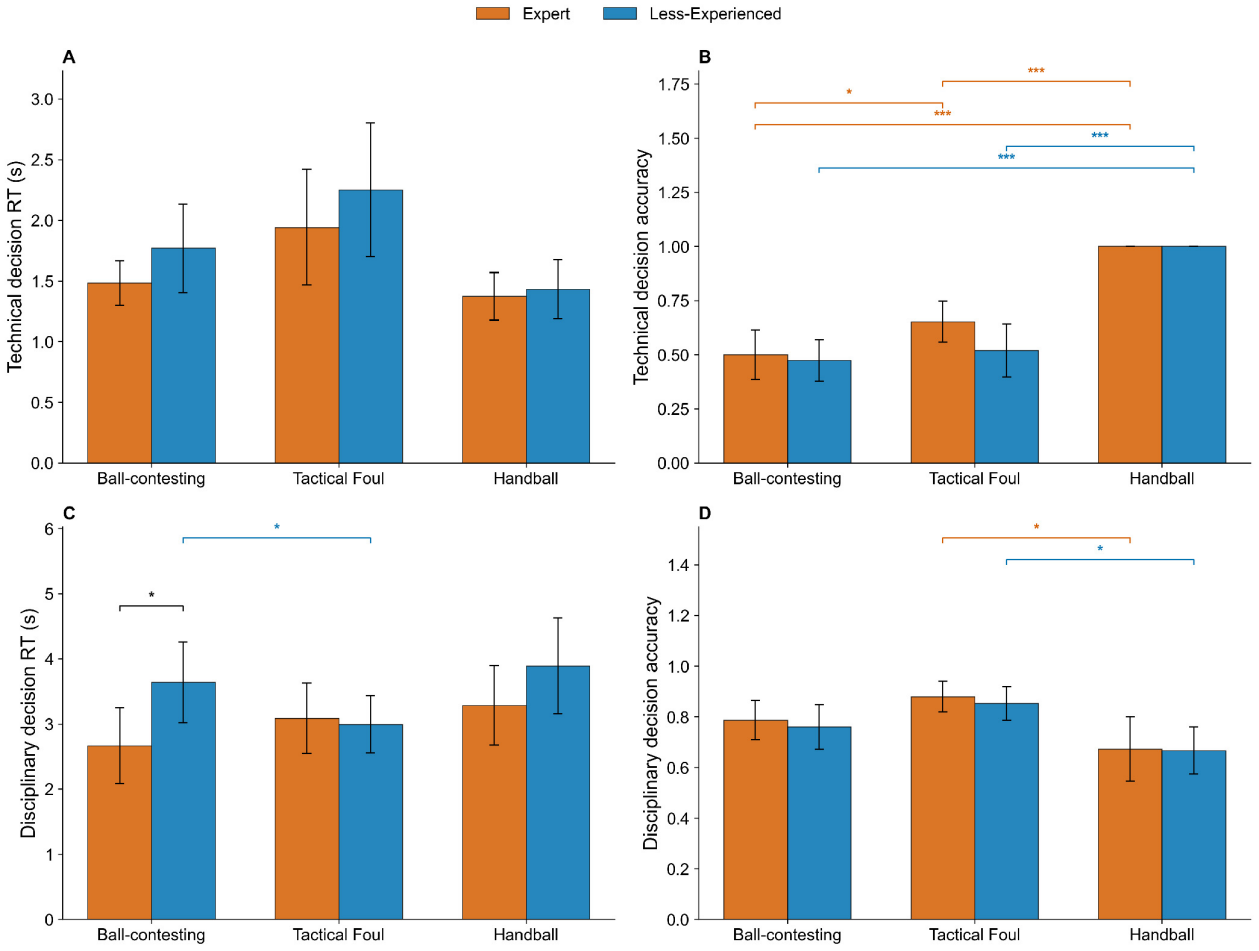
Behavioral performance of expert and less-experienced referees across three foul situations. **(A)** Technical decision RT; **(B)** technical decision accuracy; **(C)** disciplinary decision RT; **(D)** disciplinary decision accuracy. Bars show group means with 95% confidence intervals derived from the *t*-distribution (*n* = 15/group). Brackets between situations denote *post-hoc* pairwise comparisons of the situation main effect (Holm–Bonferroni corrected; orange = significant within experts, blue = significant within less-experienced referees); brackets between expert and less-experienced bars denote the group simple effect within that situation. ^*^p < 0.05, ^**^p < 0.01, ^***^p < 0.001.

Descriptive statistics for technical and disciplinary decisions are presented in Tables 1, 2. For technical decisions, the situation main effect on accuracy was significant, *F*_(2, 56)_ = 116.449, *p* < 0.001, $\eta_{p}^{2}$ = 0.806; *post-hoc* comparisons indicated that accuracy in handball was significantly higher than in tactical foul and ball-contesting in both groups. The group main effect was non-significant, *F*_(1, 28)_ = 1.719, *p* = 0.200, $\eta_{p}^{2}$ = 0.058, as was the Group × Situation interaction, *F*_(2, 56)_ = 1.957, *p* = 0.151, $\eta_{p}^{2}$ = 0.065. Reaction times for technical decisions exhibited the same situation pattern, *F*_(1.37, 38.22)_ = 11.367, *p* = 0.001, $\eta_{p}^{2}$ = 0.289; reaction times in tactical foul situations were significantly longer than in the other two situations, with neither the group main effect nor the Group × Situation interaction reaching significance (all *p* > 0.05; Figures 2A, B).

**Table 1:** Descriptive statistics of technical decisions (*M* ± SD, *n* = 15/group).

Situation	Expert ACC	Expert RT (s)	Less-experienced ACC	Less-experienced RT (s)
Ball-contesting	0.50 ± 0.21	1.48 ± 0.33	0.47 ± 0.17	1.77 ± 0.66
Handball	1.00 ± 0.00	1.37 ± 0.36	1.00 ± 0.00	1.43 ± 0.44
Tactical foul	0.65 ± 0.17	1.94 ± 0.86	0.52 ± 0.22	2.25 ± 0.99

**Table 2:** Descriptive statistics of disciplinary decisions (*M* ± SD, *n* = 15/group).

Situation	Expert ACC	Expert RT (s)	Less-experienced ACC	Less-experienced RT (s)
Ball-contesting	0.79 ± 0.14	2.67 ± 1.05	0.76 ± 0.16	3.64 ± 1.12
Handball	0.67 ± 0.23	3.29 ± 1.10	0.67 ± 0.17	3.89 ± 1.33
Tactical foul	0.88 ± 0.11	3.09 ± 0.97	0.85 ± 0.12	2.99 ± 0.80

For disciplinary decisions, accuracy showed a significant situation main effect, *F*_(2, 56)_ = 13.732, *p* < 0.001, $\eta_{p}^{2}$ = 0.329; both groups achieved significantly higher accuracy in tactical foul situations than in handball. Neither the group main effect nor the interaction reached significance (both *p* > 0.05). For reaction time, the group main effect was non-significant, *F*_(1, 28)_ = 2.311, *p* = 0.140, whereas the Group × Situation interaction was significant, *F*_(2, 56)_ = 4.158, *p* = 0.021, $\eta_{p}^{2}$ = 0.129. Simple-effect analysis indicated that experts responded faster than less-experienced referees in ball-contesting situations (*F*_(1, 28)_ = 6.071, *p* = 0.020, $\eta_{p}^{2}$ = 0.178), whereas no group differences emerged in handball or tactical foul situations (Figures 2C, D).

### 3.2 Global eye-movement behavior

Descriptive statistics for global eye-movement indices appear in Table 3. Fixation duration showed a significant situation main effect, *F*_(1.19, 33.31)_ = 16.854, *p* < 0.001, $\eta_{p}^{2}$ = 0.376; both groups showed significantly longer fixation durations in tactical foul situations than in the other two situations, with neither the group main effect nor the interaction reaching significance (all *p* > .05). Fixation count showed a significant situation main effect, *F*_(2, 56)_ = 52.012, *p* < 0.001, $\eta_{p}^{2}$ = 0.650; both groups exhibited significantly higher fixation counts in handball than in the other two situations, with no significant group or interaction effect. Saccade count followed an analogous situation pattern, *F*_(2, 56)_ = 54.613, *p* < 0.001, $\eta_{p}^{2}$ = 0.661, with non-significant group and interaction effects.

**Table 3:** Descriptive statistics of global eye-movement indices (*M* ± SD, *n* = 15/group).

Indicator	Group	Ball-contesting	Handball	Tactical foul
Fixation duration (s)	Expert	3.06 ± 0.25	3.10 ± 0.11	3.22 ± 0.05
	Less-experienced	3.14 ± 0.11	3.16 ± 0.07	3.25 ± 0.07
Fixation count	Expert	8.33 ± 1.50	9.33 ± 1.10	7.97 ± 1.02
	Less-experienced	8.57 ± 1.63	9.59 ± 1.60	7.62 ± 1.28
Saccade count	Expert	7.60 ± 1.48	8.50 ± 1.11	7.12 ± 1.04
	Less-experienced	7.74 ± 1.67	8.85 ± 1.63	6.78 ± 1.38
Pupil diameter (a.u.)	Expert	1,104 ± 364	1,060 ± 343	985 ± 341
	Less-experienced	1,173 ± 277	1,107 ± 255	1,006 ± 242

**Figure 3:**
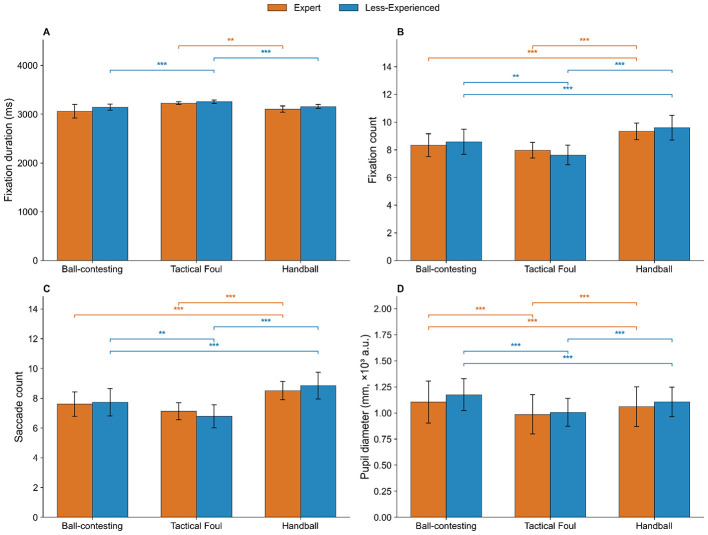
Global eye-movement behavior of expert and less-experienced referees across three foul situations. **(A)** Fixation duration; **(B)** fixation count; **(C)** saccade count; **(D)** pupil diameter (arbitrary units, ×10^3^). Bars show group means with 95% confidence intervals (*n* = 15/group). Brackets between situations denote *post-hoc* pairwise comparisons of the situation main effect (Holm–Bonferroni corrected; orange = within experts, blue = within less-experienced referees). Within-situation group simple effects did not reach significance and are therefore not shown. ^*^p < 0.05, ^**^p < 0.01, ^***^p < 0.001.

Pupil diameter showed a significant situation main effect, *F*_(1.38, 38.72)_ = 165.581, *p* < 0.001, $\eta_{p}^{2}$ = 0.855; both groups showed significantly smaller pupil diameters in tactical foul situations than in the other two. The Group × Situation interaction also reached significance, *F*_(1.38, 38.72)_ = 4.653, *p* = 0.026, $\eta_{p}^{2}$ = 0.142; however, simple-effect analyses indicated that within-situation group differences in pupil diameter did not reach significance (all *p* > 0.05), and the interaction reflected differences between the two groups in the magnitude of pupil change across situations (Figure 3).

### 3.3 AOI-level eye-movement behavior

**Figure 4:**
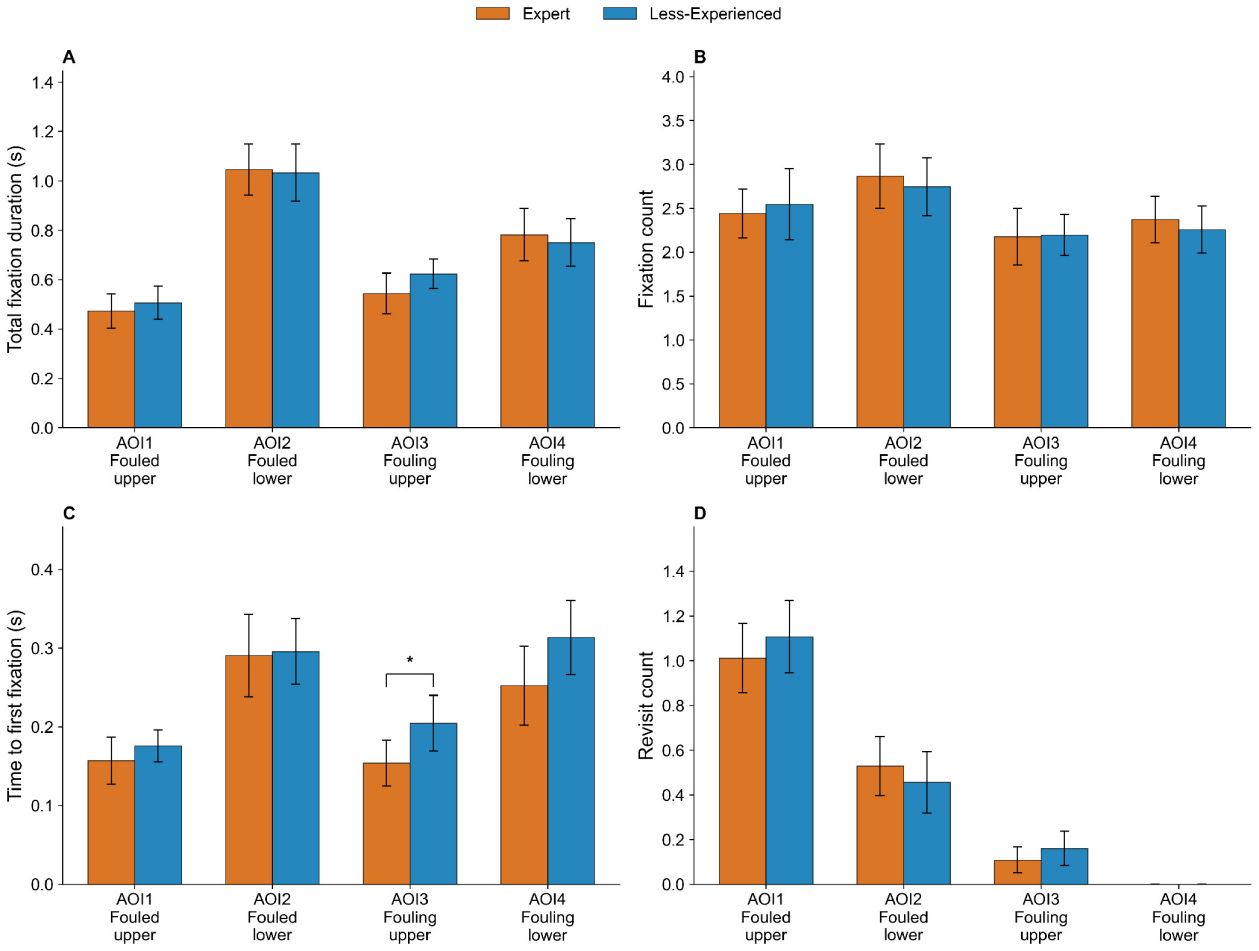
AOI-level eye-movement behavior in ball-contesting situations. **(A)** Total fixation duration; **(B)** fixation count; **(C)** time to first fixation; **(D)** revisit count. Bars show means with 95% confidence intervals (*n* = 15/group). ^*^ above the bracket indicates a significant independent-samples *t*-test between expert and less-experienced at that AOI (*p* < 0.05).

In ball-contesting situations, descriptive statistics for total fixation duration, fixation count, time to first fixation and revisit count across AOIs, together with the corresponding independent-samples *t*-tests, are presented in Table 4. Total fixation duration, fixation count and revisit count did not differ significantly between groups across any AOI. The time to first fixation differed significantly at AOI3 (the upper body of the fouling player), with experts showing shorter TFF than less-experienced referees, *t*_(26.02)_ = −2.38, *p* = 0.024, *d* = −0.87 (Figure 4).

**Table 4:** Ball-contesting AOI-level eye-movement indices and between-group comparisons (*M* ± SD, *n* = 15/group).

AOI	Group	TFD (s)	Fix. count	TFF (s)	Revisits
AOI1 (fouled, upper body)	Expert	0.47 ± 0.13	2.44 ± 0.50	0.16 ± 0.06	1.01 ± 0.28
	Less-experienced	0.51 ± 0.12	2.55 ± 0.74	0.18 ± 0.04	1.11 ± 0.29
AOI2 (fouled, lower body)	Expert	1.05 ± 0.19	2.87 ± 0.66	0.29 ± 0.09	0.53 ± 0.24
	Less-experienced	1.03 ± 0.21	2.75 ± 0.60	0.30 ± 0.08	0.46 ± 0.25
AOI3 (fouling, upper body)	Expert	0.54 ± 0.15	2.18 ± 0.58	0.15 ± 0.05^*^	0.11 ± 0.10
	Less-experienced	0.62 ± 0.11	2.20 ± 0.42	0.21 ± 0.06^*^	0.16 ± 0.14
AOI4 (fouling, lower body)	Expert	0.78 ± 0.19	2.37 ± 0.48	0.25 ± 0.09	0.00 ± 0.00
	Less-experienced	0.75 ± 0.17	2.26 ± 0.49	0.31 ± 0.09	0.00 ± 0.00

TFD, total fixation duration; TFF, time to first fixation.

^*^Independent-samples *t*-test *p* < 0.05. AOI-level comparisons were exploratory and were not corrected for multiple comparisons.

**Figure 5:**
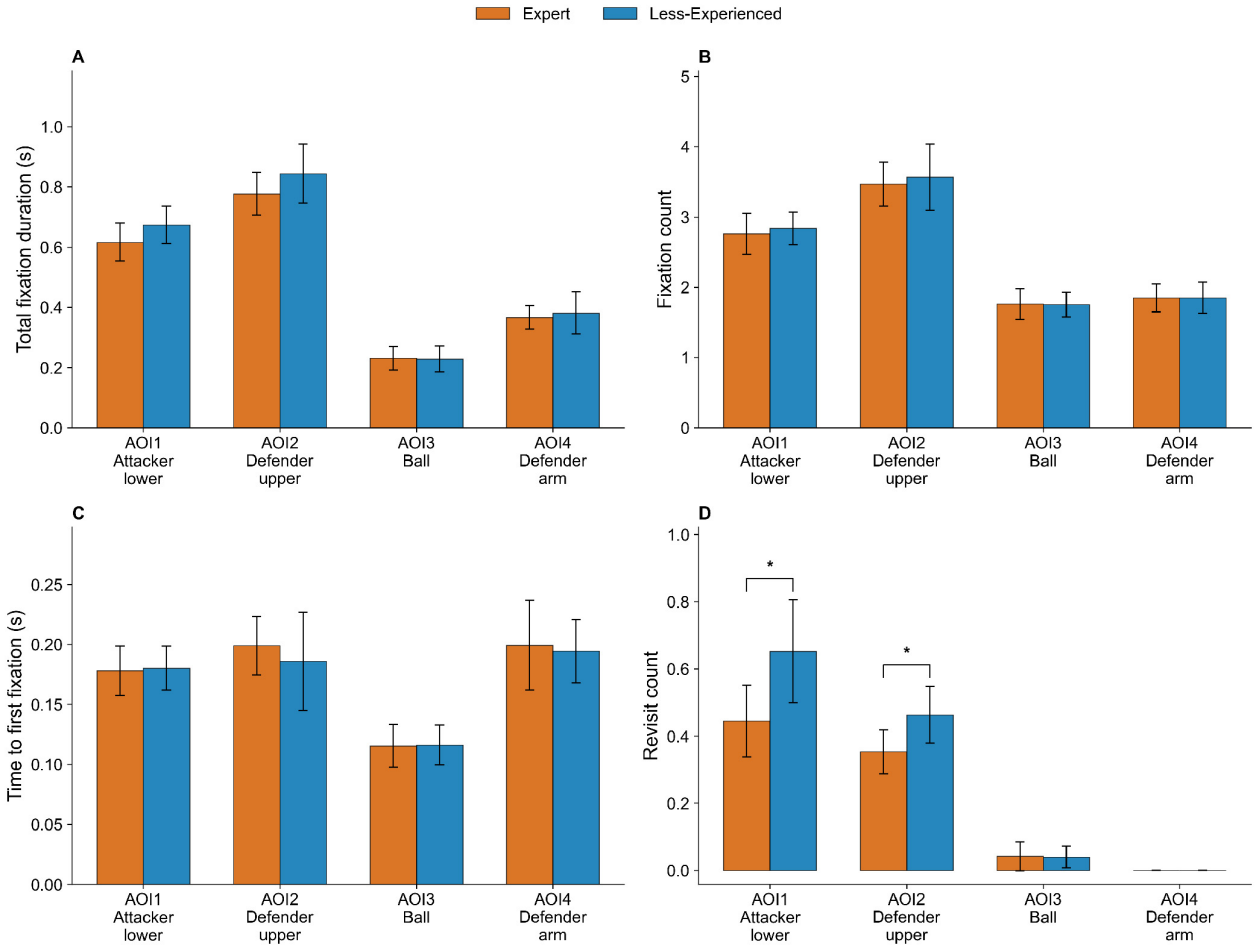
AOI-level eye-movement behavior in handball situations. **(A)** Total fixation duration; **(B)** fixation count; **(C)** time to first fixation; **(D)** revisit count. Bars show means with 95% confidence intervals (*n* = 15/group). ^*^ above the bracket indicates a significant independent-samples *t*-test between expert and less-experienced at that AOI (*p* < 0.05).

For handball situations, the corresponding descriptive statistics and independent-samples *t*-tests are presented in Table 5. Total fixation duration, fixation count and time to first fixation showed no significant between-group differences across any AOI. Revisit count differed significantly at AOI1 (the lower body of the attacking player) and AOI2 (the upper body of the defending player), with experts showing fewer revisits than less-experienced referees in both cases: *t*_(24.98)_ = −2.38, *p* = 0.025, *d* = −0.87, and *t*_(26.31)_ = −2.20, *p* = 0.037, *d* = −0.80, respectively (Figure 5).

**Table 5:** Handball AOI-level eye-movement indices and between-group comparisons (*M* ± SD, *n* = 15/group).

AOI	Group	TFD (s)	Fix. count	TFF (s)	Revisits
AOI1 (attacker, lower body)	Expert	0.62 ± 0.11	2.76 ± 0.53	0.18 ± 0.04	0.44 ± 0.19^*^
	Less-experienced	0.67 ± 0.11	2.84 ± 0.41	0.18 ± 0.03	0.65 ± 0.28^*^
AOI2 (defender, upper body)	Expert	0.78 ± 0.13	3.47 ± 0.56	0.20 ± 0.05	0.35 ± 0.12^*^
	Less-experienced	0.84 ± 0.18	3.57 ± 0.85	0.19 ± 0.07	0.46 ± 0.15^*^
AOI3 (ball)	Expert	0.23 ± 0.07	1.76 ± 0.40	0.12 ± 0.03	0.04 ± 0.08
	Less-experienced	0.23 ± 0.08	1.75 ± 0.32	0.12 ± 0.03	0.04 ± 0.06
AOI4 (defender, arm)	Expert	0.37 ± 0.07	1.85 ± 0.35	0.20 ± 0.07	0.00 ± 0.00
	Less-experienced	0.38 ± 0.13	1.85 ± 0.40	0.19 ± 0.05	0.00 ± 0.00

TFD, total fixation duration; TFF, time to first fixation. ^*^Independent-samples *t*-test *p* < 0.05. AOI-level comparisons were exploratory and were not corrected for multiple comparisons.

### 3.4 Visualization of fixation heat maps and scan paths

**Figure 6:**
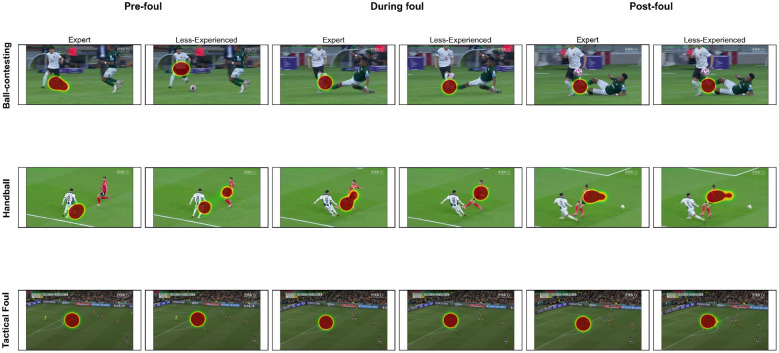
Fixation heat maps for expert and less-experienced referees pre-, during-, and post-foul across the three foul situations. Color intensity indicates fixation frequency and duration; red marks the densest regions. Source: FIFA Referee Assistance and Education (FIFA RED) platform.

Fixation heat maps and scan-path plots provide complementary qualitative observations of between-group differences in visual behavior. In ball-contesting situations, prior to the foul, experts appeared to concentrate their fixations on players' body posture, whereas less-experienced referees distributed fixations more diffusely between the lower limbs and the ball; during the foul, experts extended visual attention beyond the contact point in a more anticipatory manner, while less-experienced referees fixated mainly on the contact point itself; after the foul, both groups converged in fixating the lower limbs of the defensive and fouling players. In handball situations, prior to the foul, experts distributed fixations between the attacker's lower limbs and the defender's torso, whereas less-experienced referees focused mainly on the kicking action and ball trajectory with more dispersed fixations; during the foul, experts appeared to concentrate on the defender's arm, while less-experienced referees appeared to show delayed and less focused attention; after the foul, both groups partially tracked the ball's movement. In tactical foul situations, fixation patterns did not differ markedly between the two groups across the three phases, with both groups concentrating on the fouling and fouled players within the foul area (Figure 6).

**Figure 7:**
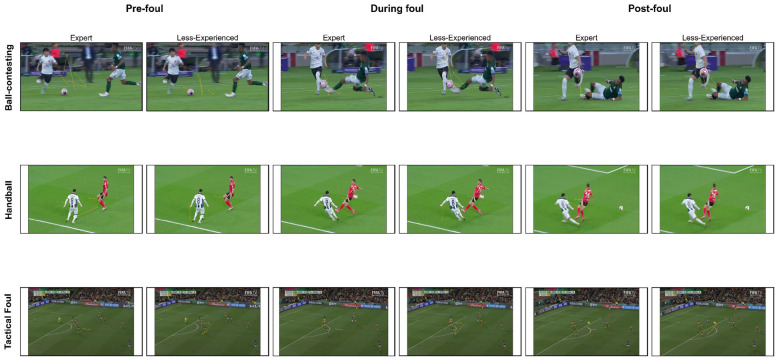
Scan paths for expert and less-experienced referees pre-, during-, and post-foul across the three foul situations. Dot size encodes fixation duration; lines indicate the saccade sequence. Source: FIFA Referee Assistance and Education (FIFA RED) platform.

Scan-path visualization suggested that experts appeared to produce more structured and continuous saccade sequences in ball-contesting and handball situations. In ball-contesting situations, experts' fixations traversed the relevant body regions of both players in turn, whereas less-experienced referees tended to show comparatively scattered and discontinuous trajectories; in handball situations, experts' fixation sequences progressed from the attacker's lower limbs through the torso to the defender's arm, whereas less-experienced referees repeatedly inspected multiple regions and exhibited greater uncertainty. In tactical foul situations, experts appeared to focus on the position of the last and second-to-last defenders prior to and during the foul, with less-experienced referees showing shorter fixations and weaker directional structure; after the foul, experts continued monitoring defensive positioning, while less-experienced referees fixated mainly around the penalty arc. All visualization results are qualitative observations and have not undergone quantitative testing (Figure 7). These qualitative visualization patterns should be interpreted cautiously and require future quantitative validation using dedicated scan-path analysis methods such as Scan Match.

## 4 Discussion

The provenance of refereeing decision performance has long been a focus of empirical research. Accumulated evidence indicates a stable relationship between officiating experience and decision accuracy, but the causal chain is not unitary. Decision accuracy may be shaped by both rule-knowledge mastery and information-extraction efficiency, as well as by cognitive-resource allocation (Mann et al., 2007; Spitz et al., 2016). The present comparison of national-level and Class-3 football referees across three foul situations indicates that expertise-related differences are unevenly distributed across the dimensions of accuracy and efficiency, and that this distribution is sensitive to situation type.

Decision accuracy did not differ significantly between groups for either technical or disciplinary judgements, an outcome consistent with the direction of training-intervention research. Structured judgement tasks rely on declarative rule knowledge that can be acquired through systematic video-based training and assessment cycles (Catteeuw et al., 2009; Put et al., 2013; Zhou et al., 2025); after passing certification examinations and basic training, Class-3 referees may already approach national-level performance on most standardized tasks. The 100% accuracy in handball situations across both groups likely reflects relatively explicit judgement criteria and concentrated visual cues, compressing decision difficulty to a ceiling level; conversely, near-chance accuracy in ball-contesting situations across both groups suggests greater contextual ambiguity, requiring subjective judgement of contact intensity and intent, with rule knowledge offering only marginal advantage (Spitz et al., 2016). The graded accuracy across situations is consistent with the initial expectation that expertise levels would not differentiate strongly on rule-bound tasks, and suggests that decision accuracy alone may not provide a reliable index of officiating expertise.

Expertise-related differences emerged more clearly in efficiency-related indices, although this manifestation was situation-specific. In ball-contesting situations, experts' disciplinary reaction times were significantly shorter than those of less-experienced referees, and simple-effect analyses confirmed that this difference was indeed significant within this situation. Multisource information integration in ball-contesting carries a high demand, and the rule-defined judgement boundary is comparatively diffuse; under such conditions, the pattern-recognition mechanisms accumulated through experience reduce decision cost more effectively (Chase and Simon, 1973; Mann et al., 2007). Pupil diameter showed a significant Group × Situation interaction, although within-situation simple effects did not reach significance; the interaction is therefore best interpreted as reflecting differences between the two groups in the magnitude of pupil change across situations rather than as a within-situation group difference (van der Wel and van Steenbergen, 2018). It should be noted, however, that pupil diameter can also be influenced by low-level visual properties of the stimuli, such as luminance and contrast. Although the present findings are broadly consistent with differences in cognitive resource allocation, this alternative explanation cannot be fully ruled out and should be considered when interpreting the results. In addition, the video clips were not formally matched for luminance or contrast across situations, and no baseline correction was applied to the pupillometry data. The interaction pattern remains broadly consistent with the interpretation that experts regulate cognitive resources with greater sensitivity across situations, but verification awaits larger samples and additional neurophysiological measures (Nalçacıgil et al., 2025). Overall fixation duration, fixation count and saccade count did not differ between groups, indicating that expert advantages do not arise from looking more or looking longer but rather from the directionality of fixation allocation (Hancock and Ste-Marie, 2013; Williams and Davids, 1998).

AOI-level analyses provided more direct evidence of this directionality. In ball-contesting situations, experts' time to first fixation on the upper body of the fouling player was significantly shorter than that of less-experienced referees; this region encodes critical judgement cues such as movement intent and body posture (Spitz et al., 2016). In handball situations, experts revisited the attacker's lower body and the defender's upper body significantly less often than less-experienced referees, suggesting that experts complete the necessary information integration during the initial fixation and need not re-inspect secondary AOIs to reduce uncertainty (Savelsbergh et al., 2005). The qualitative scan-path visualization aligns with this statistical pattern: experts appeared to produce more structured and goal-directed saccade sequences, whereas less-experienced referees showed more dispersed and repetitive search patterns (Williams and Davids, 1998). These observations are consistent with the theoretical expectation of top-down attention regulation: when task knowledge and accumulated experience consolidate into a stable internal template, attention allocation can be template-driven rather than stimulus-salience-driven (Brimmell et al., 2024; Corbetta and Shulman, 2002). It should be noted that AOI-level differences emerged in only 3 of 56 comparisons; these results should therefore be treated as exploratory and validated through preregistered tests in future research (Mann et al., 2007; Moore et al., 2019).

The findings carry direct implications for referee training. When decision accuracy in structured tasks is already brought close to ceiling by rule-based training, the marginal-gain space of training systems likely lies more in efficiency and attention allocation than in further accuracy improvements. Situation-driven perceptual–cognitive training combined with AOI-based eye-movement feedback and high-fidelity video or virtual-reality simulation may help trainees build the fixation-allocation templates characteristic of high-level officiating (Helsen et al., 2023; Put et al., 2013; van Biemen et al., 2023; Zhou et al., 2025). The findings likewise suggest that single-indicator evaluation (such as accuracy alone) is insufficient for fully appraising officiating performance; incorporating reaction time, pupillometry and AOI temporal indices into evaluation systems may provide a more sensitive yardstick for selection and assessment.

## 5 Limitations and future directions

Several limitations should be acknowledged. The video-based task supports standardization and between-group comparison but cannot reproduce the physical, emotional, spatial-locomotion and social pressures of real-match contexts (Lopes de Lima et al., 2025; van Biemen et al., 2023; van Biemen and Mann, 2024). Therefore, the present laboratory-based task captures only part of the perceptual–cognitive and contextual demands involved in real-match officiating. Moreover, real-world refereeing requires active positioning and continuous movement to obtain optimal two- and three-dimensional viewing angles. Because the present study used fixed-angle broadcast footage, participants could not utilize dynamic positioning strategies that may contribute to expert officiating performance in live matches. The sample size, constrained by the population of national-level referees, leaves the test of the group main effect underpowered for small-to-medium effects; the stimulus set, though covering three typical foul situations, contained only 10 clips per situation and cannot exhaust the situational diversity of real matches (Samuel et al., 2024); and the AOI-level multiple comparisons were not corrected for the family-wise error rate, so the corresponding results should be treated as exploratory. The ceiling effect in handball and the near-chance accuracy in ball-contesting indicate that part of the stimulus difficulty distribution lies at the extremes; future work should refine difficulty pre-screening. Future research should incorporate larger and cross-tier samples, real-match or virtual-reality officiating tasks, and multimodal physiological measures such as electroencephalography and heart-rate variability, to test more directly whether the efficiency advantages observed here remain robust under conditions of higher ecological validity (van Biemen and Mann, 2024).

## 6 Conclusions

In a video-based judgement task, national-level and Class-3 football referees did not differ substantively on decision accuracy, but a directional pattern consistent with expertise emerged for disciplinary reaction times in ball-contesting situations and for several AOI-level visual-search indices. The findings indicate that the advantages accumulated through officiating experience are, in the present setting, manifested primarily in efficiency-related indices and exhibit situation specificity; the observation is compatible with the theoretical expectation of top-down attention regulation but requires further validation in designs of higher ecological validity and larger sample size. In refereeing selection and training, incorporating efficiency and attention-allocation indices into evaluation and training systems may offer a more sensitive developmental pathway than reliance on decision accuracy alone.

## Data Availability

The raw data supporting the conclusions of this article will be made available by the authors, without undue reservation.
